# The 3D8 single chain variable fragment protein suppresses Newcastle disease virus transmission in transgenic chickens

**DOI:** 10.1186/s12917-020-02462-9

**Published:** 2020-08-06

**Authors:** Sung June Byun, Hoonsung Choi, Shanmugam Sureshkumar, Seong-Su Yuk, Jung-Hoon Kwon, Jin-Yong Noh, Sun Keun Jung, Jeom Sun Kim, Keon Bong Oh, Hyeon Yang, Gunsup Lee, Hwi-Cheul Lee, Jae-Seok Woo, Chang-Seon Song

**Affiliations:** 1 Animal Biotechnology Division, National Institute of Animal Science, Rural Development Administration, 55365 Wanju-gun, Republic of Korea; 2 Avian Disease Laboratory, College of Veterinary Medicine, Konkuk University, 1 Hwayang-dong, Gwangjin-gu, 05029 Seoul, Republic of Korea

**Keywords:** Newcastle disease virus (NDV), 3D8 ScFv, chicken, transgenic

## Abstract

**Background:**

The 3D8 single chain variable fragment (scFv) is a mini-antibody sequence that exhibits independent nuclease activity against all types of nucleic acids. In this research, crossing a 3D8 scFv G1 transgenic rooster with wild-type hens produced 3D8 scFv G_2_ transgenic chickens to evaluate suppression of viral transmission.

**Result:**

The transgenic chickens were identified using genomic PCR and immunohistochemistry. To evaluate Newcastle disease virus (NDV) protection conferred by 3D8 scFv expression, transgenic, non-transgenic, and specific pathogen-free (SPF) chickens were challenged with virulent NDV by direct injection or aerosol exposure. The three groups of chickens showed no significant differences (*p* < 0.05) in mean death time after being directly challenged with NDV; however, in contrast to chickens in the non-transgenic and SPF groups, chickens in the transgenic group survived after aerosol exposure. Although the transgenic chickens did not survive after direct challenge, we found that the chickens expressing the 3D8 scFv survived aerosol exposure to NDV.

**Conclusions:**

Our finding suggest that the 3D8 scFv could be a useful tool to prevent chickens from spreading NDV and control virus transmission.

## Background

Newcastle disease (ND) is an extremely important and economically devastating viral disease in poultry production in many countreis [1]. ND is characterized by high mortality and by nervous, respiratory, enteric, and reproductive infections [2]. The viruses that cause ND are classified in the family Paramyxoviridae under the genus *Avulavirus* and the species Newcastle disease virus (NDV) or avian paramyxovirus type 1 (APMV-1) [3]. NDV has a 15 kb genome consisting of six genes that encode a negative-sense, single-stranded, enveloped RNA virus. This virus has six structural proteins, including nucleoprotein (NP), phosphoprotein (P), matrix (M), fusion (F), hemagglutinin-neuraminidase (HN), and RNA-dependent RNA polymerase (L) [4], and additional nonstructural proteins V and W are encoded through RNA editing of the P gene [5]. In most countries, vaccination plays a vital role in controlling NDV [6, 7]. Although general immunizations provide excellent protection against clinical disease and mortality, they do not provide adequate protection against the virus and may not prevent ND outbreaks [8, 9]. Thus, detecting novel antiviral therapeutics for NDV and developing varieties that are resistant to viruses is expected to reduce the damage caused by disease.

The 3D8 single chain variable fragment (3D8 scFv) has shown non-specific DNA and RNA nuclease activity [10]. The 3D8 scFv is generated by using the variable heavy (VH) and variable light (VL) domains of an anti-DNA monoclonal antibody isolated from MRL-lpr/lpr mouse spleen cells. The ability of 3D8 scFv to hydrolyze nucleic acids and penetrate cells via a caveolae-lipid raft pathway has prompted its use in multiple antiviral applications [11]. For example, 3D8 scFv treatment of porcine kidney cells confers resistance to classical swine fever virus (CSFV) infection [12]. In addition, antiviral effects of the 3D8 scFv against DNA viruses in a human cell line (HeLa cells) and in mice have been demonstrated by measuring DNase activity and RNase activity [13]. Earlier data from oropharyngeal and cloacal swabs have shown that 3D8 scFv transgenic chickens in a contact delivery group exhibited viral clearance after inoculation with an avian influenza virus [14]. Studies have confirmed that the 3D8 scFv gene can be used as a potentially effective antiviral agent. The objective of this study was to investigate the antiviral effect of the 3D8 scFv against NDV. In this study, we produced, confirmed 3D8 scFv expressing transgenic chickens. The antiviral effect of the 3D8 scFv was evaluated via direct challenge and aerosol exposure to the virulent Kr005 strain of NDV.

## Results

### Production of transgenic chickens expressing the 3D8 scFv

**Fig. 1:**
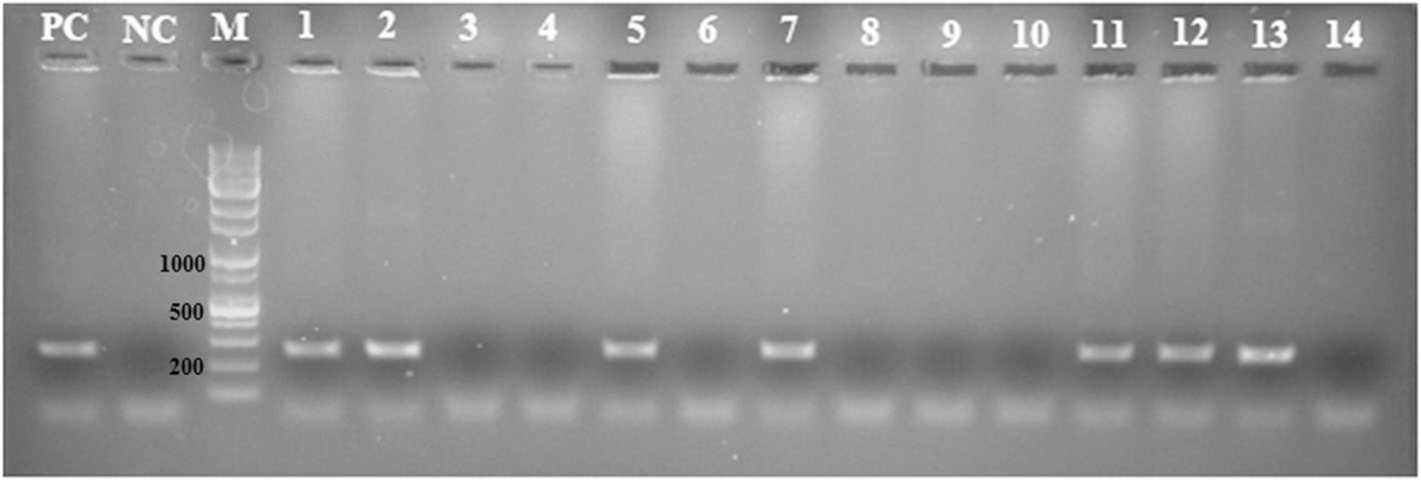
Confirmation of 3D8 scFv gene expression in G_2_ tg. Genomic PCR analysis of the G_2_ 3D8 scFv tg progeny chickens. The PCR product size is 270 bp. M: 1 kb Plus DNA ladder (SolGent), NC: negative control, PC: positive control

**Fig. 2:**
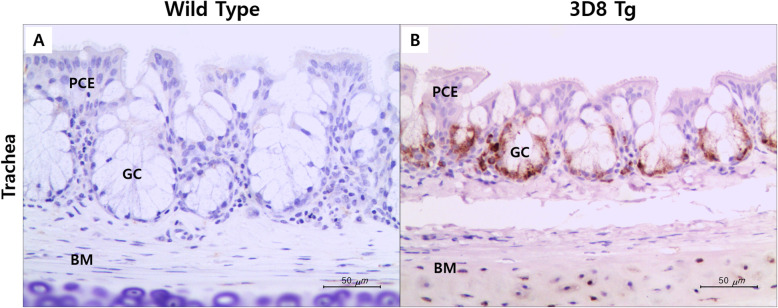
Immunohistochemistry of the tracheal tissues against 3D8 scFv from wild-type (**a**) and 3D8 transgenic chickens (**b**). The tracheal tissue of the 3D8 scFv transgenic chicken exhibited a dark brown color after DAB staining. The 3D8 transgenic chicken showed stronger staining of the lymphocytes of the tracheal lamina propria surface. PCE, pseudostratified columnar ciliated epithelium; GC, goblet cells; BM, basement membrane. The scale bar represents 50 µm. 400X

In a previous study, the CBA (chicken β-actin promoter)-3D8 scFv-HA-IRES-puro lentiviral vector was constructed and used to transfect chickens to establish G_0_ transgenic chickens [15]. In the current research, G_2_ transgenic chickens were generated by breeding a G_1_ transgenic rooster and wild-type hens for experiments on antiviral treatments against NDV. To assess 3D8 scFv expression in G_2_ transgenic chickens, genomic PCR was used (Fig. 1, supplementary Fig. 1). PCR results showed that the 3D8 scFv was present in seven of the fourteen G_2_ transgenic chickens. The ratio of transgenic to wild-type progeny was 50% in G_2_ transgenic chickens, similar to the ordinary mendelian ratio. In addition, 3D8 scFv expression in transgenic and wild-type chicken tracheal tissues was identified by immunohistochemical staining. Tracheal tissue of 3D8 scFv transgenic chickens showed scFv expression after DAB staining (Fig. 2b). Immunohistochemical staining revealed that the 3D8 scFv protein was highly expressed in lymphocytes on the surface of the tracheal lamina propria in transgenic chickens. In wild-type chickens, however, the 3D8 scFv was not observed (Fig. 2a).

### The 3D8 scFv showed antiviral activity against NDV infection in transgenic chickens

**Fig. 3:**
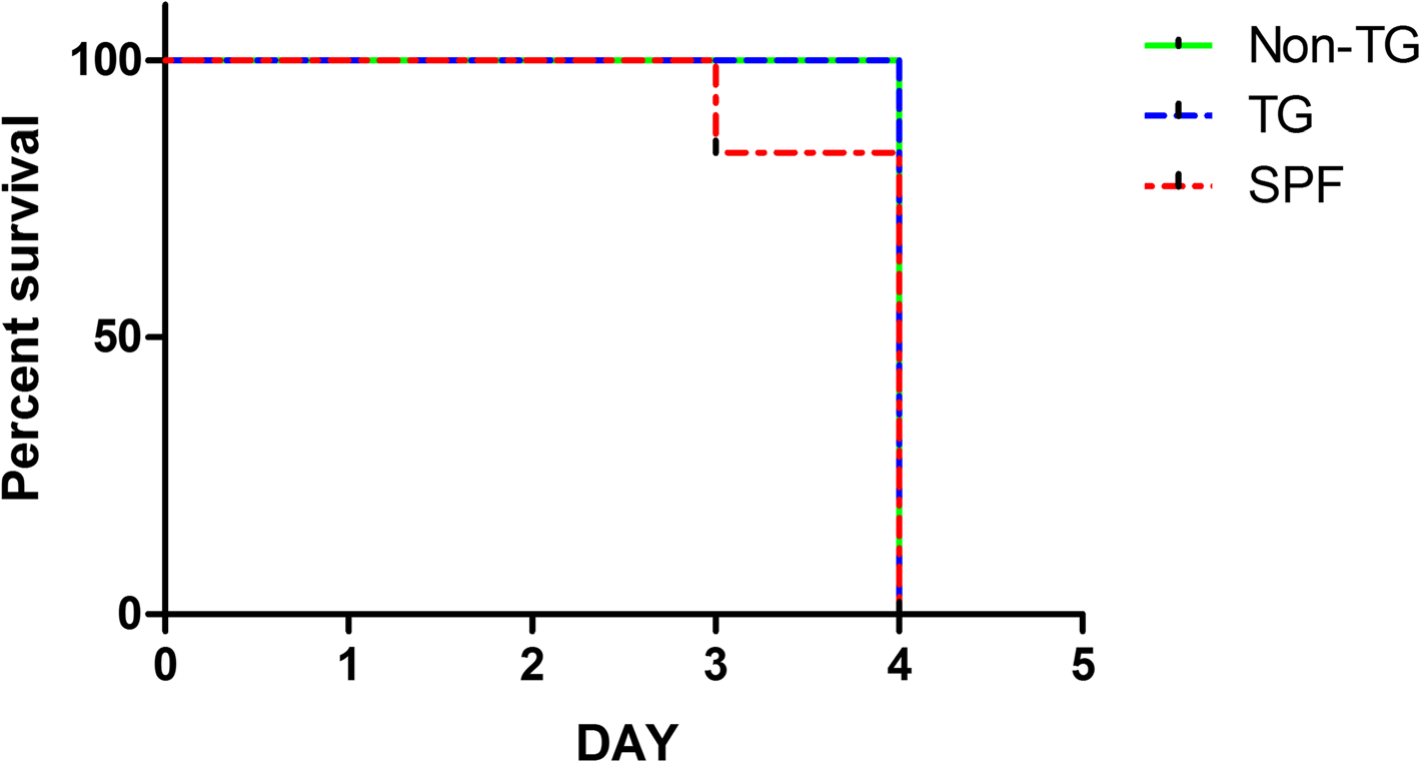
The survival rates of chickens directly challenged with NDV. Six chickens per group were directly challenged by intramuscular injection with 1 ml of 10^5.5^ EID_50_/ml of velogenic NDV, Kr005 strain. Chickens were monitored daily for 5 days to measure the clinical signs and mortality rate

**Fig. 4:**
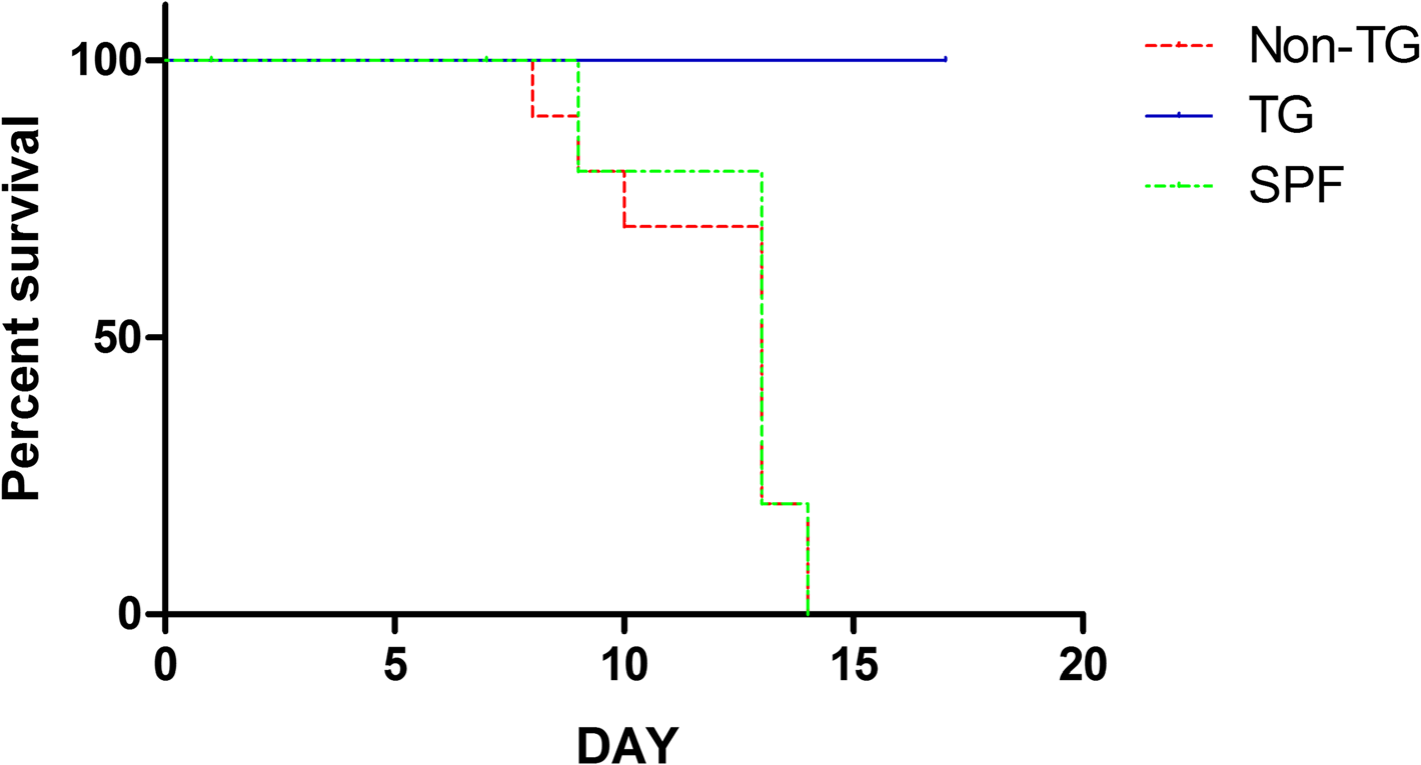
The survival rate of chickens subjected to aerosol exposure to NDV. Ten chickens per group were housed in an aerosol exposure cage with unidirectional airflow from a direct challenge cage. The cages were connected with hoses (70 cm long, 10 cm diameter, 1.5 m^2^/sec airflows). Chickens were monitored daily for 14 days to measure the clinical signs and mortality rate

We verified the antiviral impact of the 3D8 scFv against NDV infection in transgenic chickens. There were three groups of chickens: transgenic, non-transgenic, and SPF chickens. Six chickens from each group were directly infected with NDV via intramuscular injection of the virus. Cage of ten chickens per each group were connected with the cage of directly challenged chickens through a hose with airflow to determine viral transmission by exposure of the virus to chickens via aerosol. During the experimental periods, chickens were observed daily for clinical signs and mortality. NDV directly challenged chickens showed clinical signs including lethargy and depression and there was no significant difference in mean death time among groups (*p* < 0.05) (Table 1; Fig. 3). However, among the aerosol exposure groups, only the 3D8 scFv transgenic chickens survived after aerosol exposure to NDV, whereas the non-transgenic and SPF chickens showed clinical signs and died after aerosol exposure (Table 2; Fig. 4).

**Table 1:** Mortality rate of the direct challenge group for 5 days after inoculation with NDV virus

Group	No. of Chickens	Mortality ^a^	MDT^b^
Transgenic	6	6/6	4
Non-transgenic	6	6/6	4
SPF	6	6/6	3.8

^a^Mortality/Test number

^b^MDT, mean death time

**Table 2:** The mortality ratio of the indirect challenge group for 14 days after inoculation with NDV

Group	No. of Chickens	Mortality ^a^	MDT^b^
Transgenic	10	0/10	-
Non-transgenic	10	10/10	12
SPF	10	10/10	12.4

^a^Mortality/Test number

^b^MDT, mean death time

## Discussion

ND is extremely contagious and readily spreads from bird to bird. The infection is generally spread through direct contact with afflicted birds or asymptomatic birds that carry the virus, and airborne transmission is also significant [14, 16]. In particular, feathers that carry the virus can be transferred to other hosts through aerosols, suggesting that aerosol exposure is a crucial source of virus spread [17]. In addition, NDV infection may remain in a subclinical condition in immunized chickens [18, 19].

Commercial vaccination is the most significant process for the control and prevention of NDV. Inactivated and live ND vaccines are currently accessible worldwide [20]. However, it is important to understand the NDV maintenance mechanism. Experiments have shown that vaccines do not prevent replication by highly virulent viruses [6]. Many antiviral studies on NDV have taken conducted in response to these problems. Quantity of shedding, protection against mortality and clinical signs will depend on the immunity of the host. In this study, we demonstrated that expression of the 3D8 scFv in transgenic chickens could induce protection against aerosol exposure to NDV via direct antiviral effects of the 3D8 scFv, as was reported in our recent study [13, 15].

The 3D8 scFv is a mini-antibody that hydrolyzes nucleic acids without sequence specificity and is expected to prevent viral replication [10]. The 3D8 scFv has antiviral activity against a broad range of viruses, including CSFV, pseudorabies virus (PRV), herpes simplex virus (HSV), murine norovirus (MNV) and H1N1 influenza virus [12, 13, 21]. Our results also confirmed that 3D8 scFv-expressing transgenic chickens could survive an indirect challenge via aerosol exposure to NDV. In our research, we generated a 3D8 scFv G_1_ transgenic rooster with pLenti-CBA − 3D8 scFv-HA-IRES-puro vector that was mated with wild-type hens to produce G_2_ transgenic chickens. Similarly, we produced G_2_ transgenic chickens for experiments on antiviral activity against NDV by mating a G_1_ transgenic rooster and wild-type hens. Genomic PCR analysis was performed to verify that the G2 transgenic chickens expressed the 3D8 scFv. In addition, we conducted immunohistochemical staining to confirm expression of the 3D8 scFv protein in G_2_ transgenic chickens. The transgenic chickens showed high 3D8 scFv expression in lymphocytes on the tracheal lamina propria surface.

In previous study, expression of the 3D8 scFv in transgenic chickens showed that the 3D8 scFv is responsible for damaging the host genome and inducing apoptosis because the 3D8 scFv exhibits nuclease activity [15]. However, the lowest level of 3D8 scFv expression was tolerated by host cells and did not influence the expression of housekeeping RNAs, including β-actin mRNA [11]. In our previous study, we also stated that the transfected cells did not undergo apoptosis and that low levels of 3D8 scFv protein expression that did not induce apoptosis were expected in chickens. In this study, we developed G_2_ transgenic chickens expressing the 3D8 scFv and no mortality was observed in the aerosol transmission group of 3D8 scFv transgenic chickens. However, no significant effects of 3D8 scFv expression were found in the direct infection groups (p < 0.05). Since a low level of 3D8 scFv expression can exhibit antiviral activity and weakens viral infectivity in chickens, this result suggests that 3D8 scFv transgenic chickens could be useful to prevent the spread of NDV in chickens [15].

## Conclusions

In conclusion, the result suggests that the 3D8 scFv protein potentially inhibits Newcastle disease virus spreading in chicken. Therefore, the 3D8 scFv is an effective antiviral protein that should be considered as a future alternative in the poultry industry. However, there are few reports on the antiviral effect of the 3D8 scFv protein in chickens, and thus, further scientific investigations are needed.

## Methods

### Generating 3D8 scFv transgenic chickens

In a prior study, we described a 3D8 scFv G_1_ transgenic rooster which was generated with the pLenti-CBA − 3D8 scFv-HA-IRES-puro vector and mated this rooster with wild-type hens to produce G_2_ transgenic chickens [15]. PCR analysis (50 µl volume) was performed using the Phusion Blood Direct PCR Kit (Thermo Scientific). To detect the 3D8 scFv in the G_2_ chickens, we performed PCR analysis using a forward primer (5’-CCTCTGCTAACCATGTTCATGCCTTC-3’) that anneals at the CBA promoter region and a reverse primer (5’-GCTAGTGAATGTGTATCCAGAAGCCTT-3’) that anneals at the 3D8 scFv region. The following conditions were used for this analysis: initial denaturation at 98°C for 5 min; 40 cycles of 98°C for 1 s, 62°C for 5 s, and 72°C for 20 s; and final extension at 72°C for 10 min.

### Assessing 3D8 scFv protein expression in transgenic chickens by immunohistochemical staining

Tracheal tissues were collected from transgenic and wild-type chickens and fixed in 10% PFA. Tracheal tissue samples were frozen, and processed using a cryomicrotome (Leica CM3050S, Wetzlar, Germany). The resulting 3-µm-thick segments of tracheal tissue were mounted on silane-coated slides. The tissue sections were incubated with HA-tagged (3D8) primary antibodies (1:1000 dilutions) for 12 hr at 4 °C. Biotinylated secondary antibodies were applied for 1 hr at 4 °C. Chromogen 3,3’-diaminobenzidine tetrahydrochloride (DAB) (10 min) with ABC Kit (Vector Laboratories, Burlingame, CA, USA) was used to detect expression, and hematoxylin was used for counterstaining (20 min). Finally, slides were observed under light microscopy (Leica DE/DMI6000B equipped with a Leica Microsystems CMS GmbH D-35578 Wetzlar digital camera (Leica, Germany)), and images were captured. Immunohistochemical analysis was performed according to Lee et al. (2019) [13, 22, 23].

### In vivo NDV infection and transmission studies

The challenge study with a live virus was conducted in a biosafety level 2 facility under the supervision of the Institutional Animal Care and Use Committee (IACUC) (KU 18185) of Konkuk University, South Korea. In total, forty-eight three-week-old transgenic, non-transgenic (non-TG), and specific pathogen-free (SPF) chickens were used (*n* = 16). SPF chickens were obtained from Namduck Sanitec, Korea. All chickens were confirmed to be serologically negative by HI test against NDV. HI assays were performed according to the OIE standard HI method using 4 hemagglutination units (HAU) of formalin-inactivated homologous antigen. Six chickens per group were randomly allocated and directly challenged by intramuscular injection with 1 ml of 10^5.5^ 50% egg infective dose (EID_50_)/ml velogenic NDV, Kr005 strain. Six hours later, the remaining ten chickens per group were subjected to aerosol exposure to NDV through hoses (70 cm long, 10 cm in diameter, 1.5 m^2^/sec airflow) with unidirectional airflow from the cages with directly challenged chickens by cage connection. During the experimental period, the chickens were observed daily to measure the mortality rate. All chickens were identified using individual tags; water and feed were supplied *ad libitum.* Chickens were euthanized by CO2 inhalation when they were reached a predetermined humane endpoint.

### Statistical analysis

An analysis of variance (ANOVA) and Tukey–Kramer post-hoc test were used to compare the mortality between all groups. *P*-values < 0.05 were considered to be significant.

## Supplementary information

**Additional file 1: Figure S1.** Confirmation of 3D8 scFv gene expression in G_2_ tg. Genomic PCRanalysis of the G_2_ 3D8 scFv tgprogeny chickens. The PCR product size is 270 bp. M: 1kb Plus DNA ladder (SolGent), NC: negative control, PC: positive control.

## Data Availability

The datasets used and analyzed during the current study are available from the corresponding author on reasonable request.

## Abbreviations

ND: Newcastle disease
scFv: Single chain variable fragment
PCR: Polymerase Chain Reaction
SPF: Specific pathogen-free
