# Supplementary material for: The 3D8 single chain variable fragment protein suppresses Newcastle disease virus transmission in transgenic chickens
Source: BMC Vet Res. 2020 Aug 6;16:273. doi: 10.1186/s12917-020-02462-9 (PMC7409462; doi:10.1186/s12917-020-02462-9)

**Fig 1. (Cropped gel)**


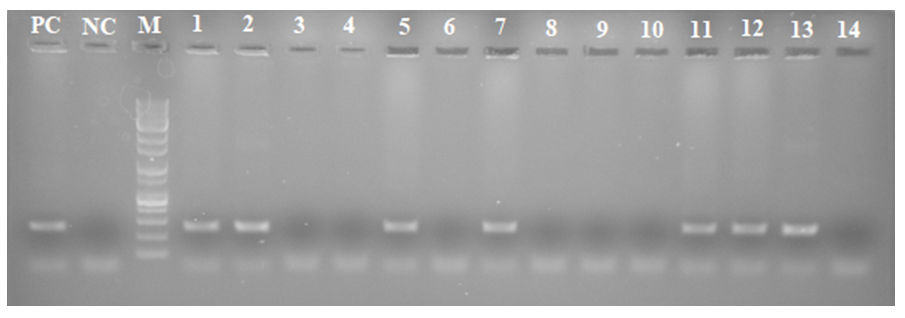


**Figure legends**

**Fig. 1**. Confirmation of 3D8 scFv gene expression in G_2_ tg. Genomic PCR analysis of the G_2_ 3D8 scFv tg progeny chickens. The PCR product size is 270 bp. M: 1 kb Plus DNA ladder (SolGent), NC: negative control, PC: positive control.

**Original Uncropped gel**


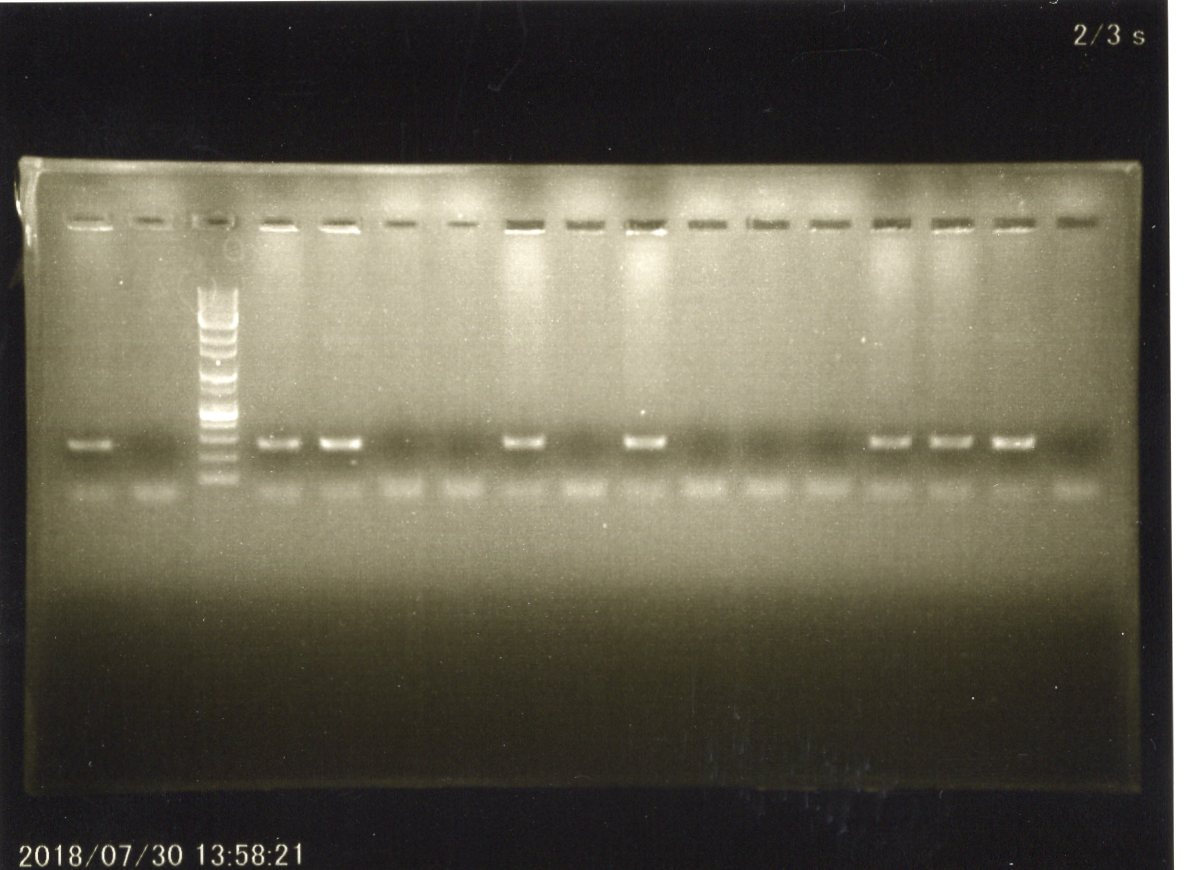

Supplement: Supplementary file 1 — Additional file 1: Figure S1. Confirmation of 3D8 scFv gene expression in G2 tg. Genomic PCRanalysis of the G2 3D8 scFv tgprogeny chickens. The PCR product size is 270 bp. M: 1kb Plus DNA ladder (SolGent), NC: negative control, PC: positive control. [file 12917_2020_2462_MOESM1_ESM.docx]
